# Lectures on Diseases of Infancy and Childhood

**Published:** 1874-08

**Authors:** 


*Lectures on the Diseases of Infancy and Childhood.* By CHARLES WEST, M. D., etc. Fifth American from the Sixth Revised and Enlarged English Edition. Philadelphia: Henry C. Lea, 1874. Buffalo: T. Butler & Son.

Dr. West's lectures on the Diseases of Children have gained a deservedly wide popularity. The standard authority in England and America, they have also been translated into German, Italian, Danish, Dutch and Russian, and a French Edition is shortly to appear.

We find many changes in this edition from the former, his modes of treatment are less active and he places more reliance upon the restorative power of nature. Most of the lectures give evidence of changes, and some have been wholly re-written, among which we notice that upon abdominal tumors, extensive changes have also taken place in the lecture on paracentesis thoracis.

The author has added largely to his number of cases since the last edition, and hence has been enabled to draw from a larger number of statistics. The formulæ have been increased somewhat, and from a cursory examination seems to be carefully arranged. The former readers and admirers of Dr. West's lectures will find many valuable and important changes in the present edition.

